# Drip Fertigation Enhances Nitrogen Uptake and Improves Winter Wheat Yield and Stability Across Planting Densities

**DOI:** 10.3390/plants15071090

**Published:** 2026-04-02

**Authors:** Xiaoyan Zhou, Mei Qian, Faming Wang, Fengjian Liang, Dapeng Gao, Shangzong Feng, Yonghui Wang, Fucheng Zhang, Xiaojun Hu

**Affiliations:** 1 College of Life Science, Linyi University, Linyi 276000, China; zhouxiaoyan@lyu.edu.cn (X.Z.); m15964386367@163.com (M.Q.); w15163901393@163.com (F.W.); gaodapeng@lyu.edu.cn (D.G.); 15762064153@163.com (F.Z.); 2 Feixian Agricultural Technology Extension Center, Feixian, Linyi 273400, China; liangfengjian@126.com; 3 Linyi Agricultural Technology Extension Center, Linyi 276001, China; taoluef@163.com; 4 Agricultural and Rural Development Center of Hedong District, Linyi 276000, China; wyh13053972999@163.com

**Keywords:** drip fertigation, planting density, source–sink coordination, nitrogen uptake, nitrogen use efficiency

## Abstract

Drip fertigation (DF) is increasingly adopted to improve winter wheat productivity, yet its interactions with planting density (PD) and the underlying source–sink mechanisms remain insufficiently quantified. Here, we evaluated winter wheat performance under two water–nitrogen (N) regimes—conventional management (CM) and DF—across a wide PD gradient (100–800 seeds m^−2^) during two growing seasons. Grain yield, yield components, population traits, dry matter production, source–sink indices, canopy N status, N uptake and N-use efficiencies were assessed. Across seasons, DF increased grain yield by 15.4–20.8% relative to CM. Yield exhibited a quadratic response to PD under both regimes; however, DF shifted the optimal PD upward (456–487 seeds m^−2^) compared with CM (377–378 seeds m^−2^) and sustained near-maximum yields over a broader PD range. DF improved population productivity by increasing productive stem percentage and grains per ear, resulting in greater grain number per m^2^ (sink size). DF also strengthened source capacity during grain filling: post-anthesis dry matter production increased by 15.5–17.6% and strongly associated with yield (*r*^2^ ≥ 0.819). Source–sink analysis suggested that DF was associated with more density treatments showing simultaneously high grain number and high post-anthesis dry matter accumulation, a pattern consistent with a broader high-yield density range. Enhanced N acquisition, especially after anthesis, may have contributed to this response. DF increased N nutrition index at anthesis and markedly increased post-anthesis N uptake by 47.7–49.5%, thereby raising total N uptake at maturity and grain N accumulation. DF improved fertilizer-N recovery efficiency and agronomic efficiency by 33.9–42.3% and 26.7–30.9%, respectively. Collectively, DF improved N uptake and source–sink coordination, enabling high yield and reduced yield penalties when planting density deviated from the optimum.

## 1. Introduction

Winter wheat is one of the most widely grown cereal crops and a cornerstone of global food security [1,2,3]. In major wheat-producing regions, sustaining high and reliable yields is increasingly challenged by variable weather, uncertainty in stand establishment, and the need to maintain strong crop growth through key developmental stages [4]. Agronomic optimization is therefore essential, because yield formation in wheat is highly sensitive to management decisions that shape canopy development, spike formation, and grain filling [5,6]. Among these decisions, planting density and water–nitrogen (N) management are two of the most influential levers: they determine population structure, resource competition within the canopy, and ultimately whether the crop can achieve high yield consistently [7,8,9,10]. Accordingly, understanding how density interacts with water–N supply strategies is fundamental for developing practical recommendations that deliver both high productivity and robust yield performance.

Planting density directly regulates yield formation through its effects on population size and individual plant productivity [11,12]. As density increases, ears per unit area typically increase, while grains per ear and grain weight often decline due to intensified competition for light, water, and nutrients [13,14]. This compensation among yield components frequently results in a unimodal (quadratic) yield response to density, with yield penalties occurring at both low density (insufficient ear number and sink capacity) and excessive density (strong intra-population competition and reduced per-plant productivity). Density also influences canopy development and crop N status: higher density accelerates canopy closure and increases LAI, but it may dilute plant N availability per unit leaf area and shorten the effective photosynthetic duration during grain filling, thereby limiting assimilate supply [15,16]. Consequently, density can affect not only yield level but also the efficiency by which applied N is captured and converted into grain, as reflected in indices such as fertilizer-N recovery efficiency (RE_N_) and agronomic efficiency (AE_N_).

Optimizing water–N management is equally critical for achieving high yield and mitigating the adverse effects of density-driven competition. Conventional practices in many intensive wheat systems rely on relatively few irrigation events combined with large basal and single topdressing N applications. Such strategies can create temporal mismatches between N supply and crop demand, leading to periods of oversupply and deficiency across the season, which may constrain ear development, accelerate senescence, and reduce grain filling—especially under dense stands. Drip fertigation (DF), which integrates irrigation and split N delivery directly into the root zone, provides an alternative approach with strong potential to support both yield formation and N acquisition [10,17,18]. By increasing the frequency of water and N supply and adjusting their proportional distribution across growth stages, DF can enhance root-zone N availability when crop demand is high, sustain canopy N status, and promote post-anthesis N uptake [19,20,21]. These mechanisms are expected to strengthen post-anthesis source capacity (assimilate supply) while supporting sink establishment (grain number), thereby improving source–sink coordination during grain filling and ultimately enhancing yield performance.

Despite the increasing adoption of DF in recent years, the interactive mechanisms between planting density and DF-driven water–N supply remain insufficiently resolved, particularly across a wide density gradient. It is unclear whether DF simply shifts the optimum density or whether it also broadens the density range that can achieve near-maximum yield by alleviating density-induced limitations on N acquisition and post-anthesis canopy function. We hypothesized that DF, by altering the number and proportion of water and N applications—especially by strengthening late-season water–N supply—can better match crop demand at higher densities. This improved alignment would maintain N availability and N uptake during critical stages (from stem elongation to anthesis and into early grain filling), sustain post-anthesis assimilate supply, and reduce the sensitivity of yield to planting density. In other words, DF is expected to broaden the planting density range over which high yield can be maintained, possibly through patterns consistent with improved source–sink matching.

To test these hypotheses, we conducted two-year field experiments comparing a drip fertigation-based management package (DF) with conventional management (CM) across eight planting densities (100–800 seeds m^−2^; PD1 = 100, PD2 = 200, PD3 = 300, PD4 = 400, PD5 = 500, PD6 = 600, PD7 = 700, PD8 = 800 seeds m^−2^) in a split-plot design. Under CM, N was applied mainly as a large basal dose with a single topdressing at jointing, and irrigation was provided by flood irrigation at three key timings. Under DF, basal N input was reduced, and the remaining N was applied through multiple fertigation events aligned with major developmental stages, together with more frequent drip irrigation. We measured grain yield and its components, population traits related to tiller survival and ear formation, dry matter production and harvest index, canopy traits at anthesis (LAI and N nutrition status), N uptake before and after anthesis, and indices of N-use efficiency. Based on these measurements, and their associations with source- and sink-related indicators such as grain number per unit area and post-anthesis dry matter accumulation, this study evaluated how the DF management package was associated with density-dependent yield formation and fertilizer-N effectiveness, and identified a practical planting density range for achieving high yield with improved N-use performance. Because multiple management components changed simultaneously under DF, the results should be interpreted as the integrated response to this treatment package rather than the isolated effect of any single factor.

## 2. Results

### 2.1. Yield Performance and Yield Components

**Figure 1:**
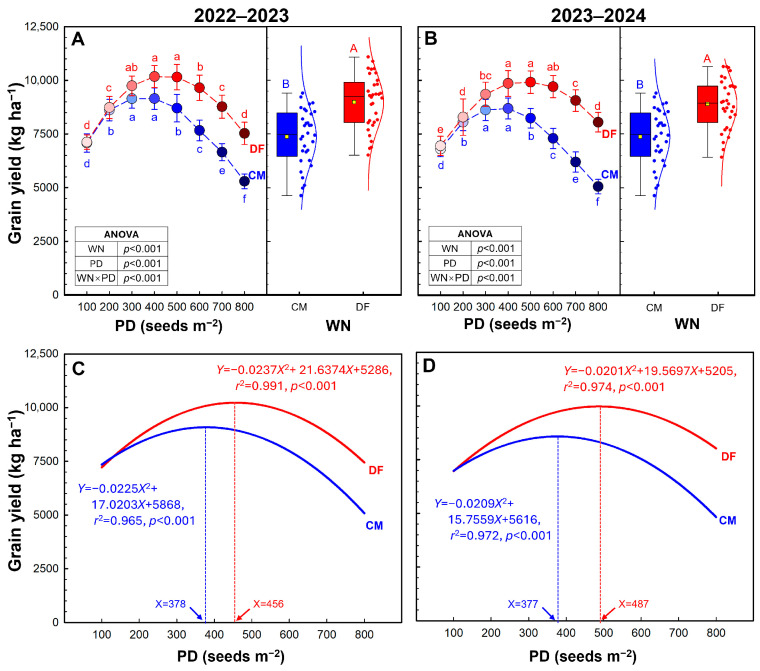
Grain yield of winter wheat (**A**,**B**) and its quadratic curve fitting (**C**,**D**) in 2022–2023 and 2023–2024 growing seasons. Data are means and error bars are SD (n = 4). Different lowercase letters indicate that there are significant differences among the planting densities (PD) at the same water-N regime (WN) according to the least significant difference test (*α* = 0.05). Different uppercase letters indicate significant differences between the means of WN across the eight PD according to the least significant difference test (*α* = 0.05). Blue and red represent CM and DF, respectively.

The grain yield was significantly affected by Water and N management regime (WN) and planting density (PD) (Figure 1A,B). Averaged across all PD, DF produced 15.4% and 20.8% higher yield than CM in 2022–2023 and 2023–2024, respectively. The grain yield increased initially and then decreased with the increasing PD under both WN regimes. In the 2022–2023 season, the highest yields of 9149 and 10,175 kg ha^−1^ were obtained at PD4 under CM and DF (Figure 1A). In the 2023–2024 season, PD5 and PD4 produced the highest yields of 8689 and 9913 kg ha^−1^ under CM and DF, respectively (Figure 1B). There is a strong quadratic regression relationship between yield and PD under both WN regimes (*r*^2^ ≥ 0.965, *p* < 0.001, Figure 1C,D). Using the fitted quadratic curves, the optimal PD under CM is 377–378 seeds m^−2^, while under DF, the optimal PD is 456–487 seeds m^−2^.

All yield components (ears per m^2^, grains per ear, and grain weight) were significantly affected by WN and PD in both seasons (Table 1). However, only the ears per m^2^ was not affected by the interaction between WN and PD. Compared to CM, DF promoted the average ears per m^2^, grains per ear, and grain weight by 6.2%, 5.5% and 2.6%, respectively, in 2022–2023. The corresponding increases were 5.7%, 4.6%, and 8.3% in 2023–2024. The ears per m^2^ increased with density, while the grains per ear and grain weight show a declining trend. The grain number per m^2^ showed a trend of first increasing and then decreasing under both WN regimes. Under CM, the maximum value was observed at PD4, while under DF, the maximum value occurred at PD5 or PD6. DF increased the average grain number per m^2^ by 12.2–13.5% relative to CM.

### 2.2. Population Size and Individual Productivity

**Figure 2:**
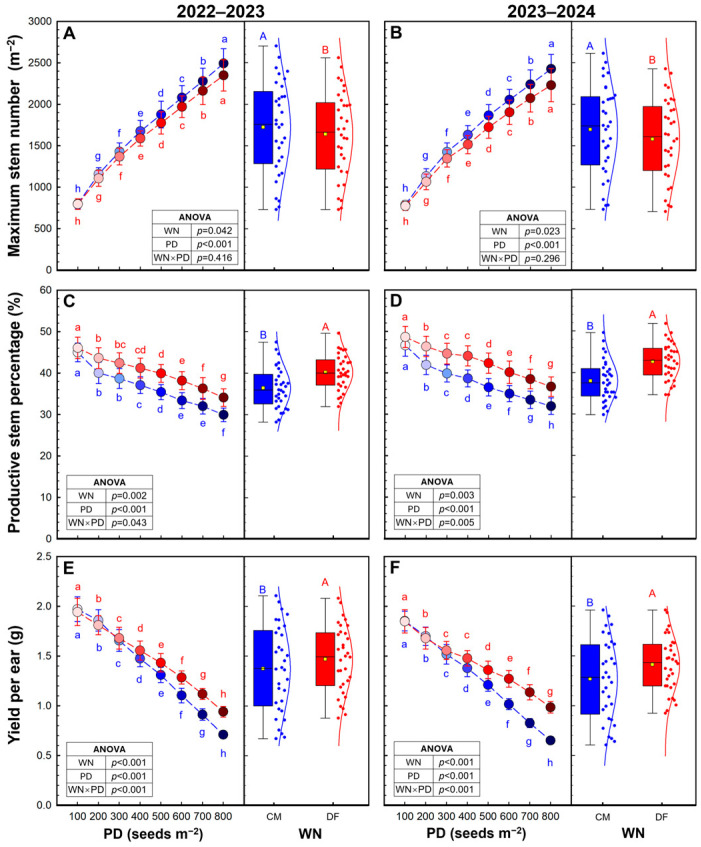
The maximum stem number (**A**,**B**), productive stems percentage (**C**,**D**) and yield per ear (**E**,**F**) in 2022–2023 and 2023–2024 growing seasons. Data are means and error bars are SD (n = 4). Different lowercase letters indicate that there are significant differences among the planting densities (PD) at the same water and N regime (WN) according to the least significant difference test (α = 0.05). Different uppercase letters indicate significant differences between the means of WN across the eight PD according to the least significant difference test (α = 0.05). Blue and red represent CM and DF, respectively.

DF had a slight negative effect on the maximum stem number (−4.9% to −6.9%) but significantly increased the productive stems percentage and yield per ear by 10.4–12.3% and 7.0–11.4% (Figure 2). DF significantly increased the productive stems percentage at any PD, except for PD1. However, the yield per ear improvement by DF was observed at PD of 400–800 seeds m^−2^.

### 2.3. Dry Matter Production and Harvest Index

Both dry matter production and harvest index were significantly affected by WN, PD, and their interaction effects (Table 2). Averaged across all planting densities, DF increased dry matter at maturity and harvest index by 10.5–11.1% and 4.5–9.7%, respectively. As planting density increased, dry matter at maturity showed a trend of first increasing and then decreasing, while harvest index continued to decrease. The peak dry matter at maturity achieved at PD4 and PD5 under CM and DF, respectively.

Compared to CM, DF promoted dry matter at anthesis and after anthesis by 7.0–8.6% and 15.5–17.6%, respectively. Dry matter at anthesis increases with planting density, but there is no clear decreasing trend. In contrast, dry matter after anthesis shows a clear trend of first increasing and then decreasing, with peak values occurring at PD3 and PD4 under CM and DF, respectively.

### 2.4. Source–Sink Coordination for High Yield Achievement

**Figure 3:**
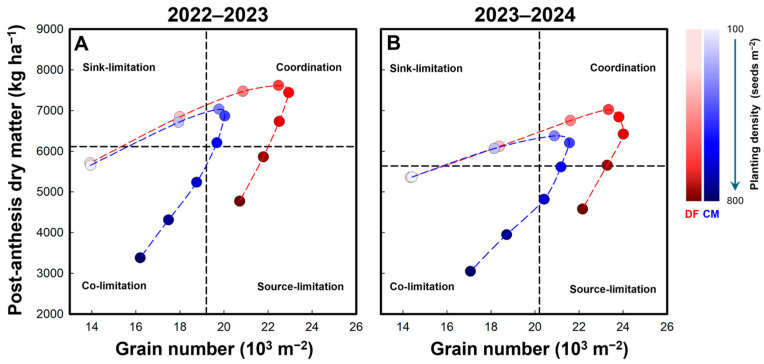
Relationship between post-anthesis dry matter accumulation and grain number under different planting densities in the 2022–2023 (**A**) and 2023–2024 (**B**) growing seasons. Vertical and horizontal dashed lines indicate the seasonal mean values of grain number and post-anthesis dry matter accumulation, respectively. The figure is intended to provide a heuristic framework for comparing treatment patterns in terms of source- and sink-related characteristics, rather than a strict physiological diagnosis based on independent biological thresholds.

The overall mean values of grain number and post-anthesis dry matter production across all treatments within each growing season were used as reference points for comparative interpretation (Figure 3). Treatments with post-anthesis dry matter production or grain number below the corresponding seasonal mean were interpreted as showing patterns consistent with relatively weaker source support or smaller sink size, respectively. This classification was intended as a descriptive and heuristic framework for comparing treatment differences, rather than as a strict physiological diagnosis based on independent biological thresholds.

Under CM, similar patterns were observed in both growing seasons. Treatments at PD1, PD7, and PD8 were located in the region characterized by relatively low grain number and low post-anthesis dry matter accumulation, indicating possible co-limitation of sink size and source support. PD2 showed a pattern consistent with relatively lower sink size, whereas the relatively high yields at PD3 and PD4 were associated with comparatively favorable source–sink matching.

Under DF, broadly similar seasonal patterns were also observed. PD2, PD8, and PD1 were located in regions suggestive of relatively lower sink size, relatively weaker source support, and possible co-limitation, respectively. In contrast, the superior yields observed at PD3–PD6 were associated with combinations of relatively high grain number and relatively high post-anthesis dry matter accumulation, a pattern consistent with improved source–sink matching under the DF management package.

### 2.5. Leaf Area Index and N Nutrition Index at Anthesis

**Figure 4:**
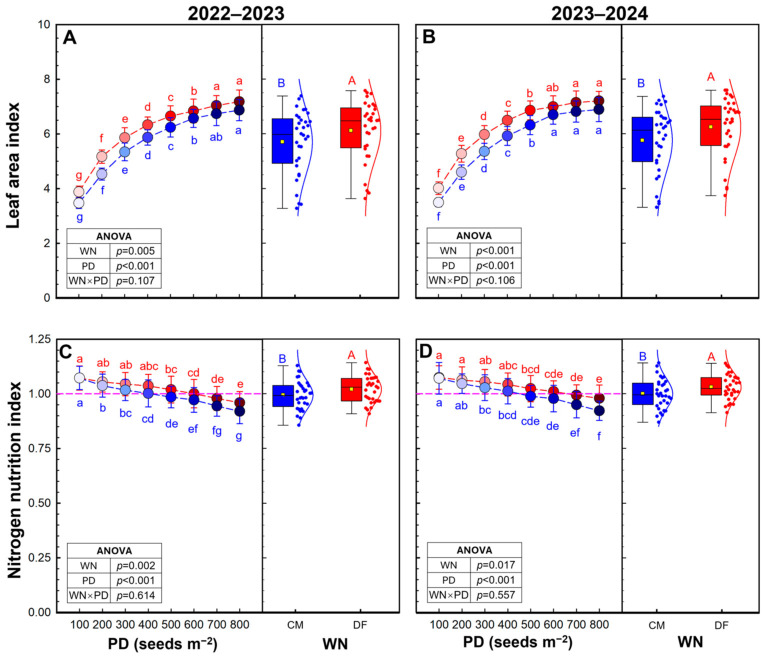
Leaf area index (**A**,**B**) and N nutrition index (**C**,**D**) of winter wheat 2022–2023 and 2023–2024 growing seasons. Data are means and error bars are SD (n = 4). Different lowercase letters indicate that there are significant differences among the planting densities (PD) at the Same water and N regime (WN) according to the least significant difference test (*α* = 0.05). Different uppercase letters indicate significant differences between the means of WN across the eight PD according to the least significant difference test (*α* = 0.05). The pink horizontal dashed line indicates NNI = 1.0. Blue and red represent CM and DF, respectively.

Two-way ANOVA indicated that both WN and PD significantly affected leaf aera index (LAI) in both seasons, while their interaction was not significant in either season (Figure 4A,B). Across densities, DF resulted in 7.3–8.4% higher LAI at anthesis than CM. LAI at anthesis increased progressively with planting density in both seasons.

Both water–N regime and planting density significantly influenced N nutrition index (NNI) in both seasons, whereas the WN × PD interaction was not significant (Figure 4C,D). Compared to CM, DF promoted the average NNI by 2.7–3.1%. In contrast to LAI, NNI at anthesis declined with increasing planting density. NNI values were generally above 1.0 at low to moderate densities but decreased progressively at higher densities, particularly under CM.

### 2.6. N Uptake and Efficiencies of N Use

N uptake at anthesis (NA), during the post-anthesis period (NPA), at maturity (NUP), and grain N accumulation (GNA) were significantly affected by WN, PD, and their interaction in both growing seasons (Table 3). Across seasons, DF consistently increased N uptake compared with CM. Averaged across densities, DF increased NA by 7.3% and 7.0%, NPA by 47.7% and 49.5%, NUP by 13.8% and 12.4%, and GNA by 15.7% and 21.4% in 2022–2023 and 2023–2024, respectively. Under both water-N regimes, NA increased from low to moderate densities and tended to plateau or slightly decline at the highest densities. In contrast, NPA showed a clear density-dependent pattern, with peak values occurring at intermediate densities (PD4–PD6 under DF), followed by declines at excessive densities. Consequently, total NUP and GNA were maximized at moderate densities (PD4–PD6), particularly under DF, where the highest NUP (296.1 and 292.8 kg ha^−1^ in 2022–2023; 287.9 and 283.9 kg ha^−1^ in 2023–2024) and GNA (222.8 and 221.5 kg ha^−1^; 221.4 and 220.9 kg ha^−1^, respectively) were observed.

**Figure 5:**
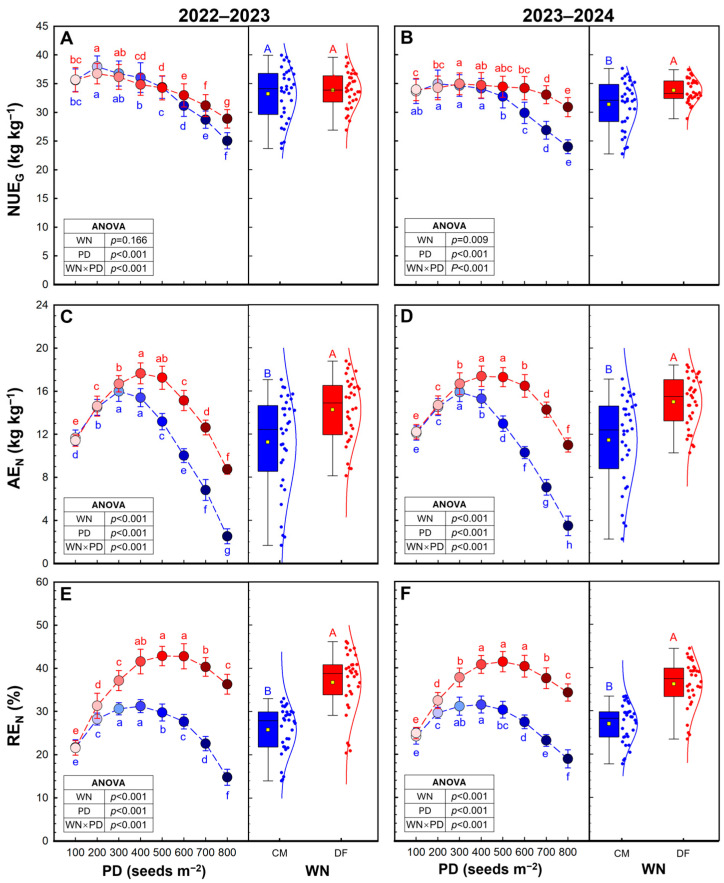
Nitrogen use efficiency for grain production (NUE_G,_ **A**,**B**), agronomic efficiency of N fertilizer (AE_N_, **C**,**D**), and recovery efficiency of N fertilizer (RE_N_, **E**,**F**) in 2022–2023 and 2023–2024 growing seasons. Data are means and error bars are SD (n = 4). Different lowercase letters indicate that there are significant differences among the planting densities (PD) at the same water and N regime (WN) according to the least significant difference test (*α* = 0.05). Different uppercase letters indicate significant differences between the means of WN across the eight PD according to the least significant difference test (*α* = 0.05).

N use efficiency for grain production (NUE_G_), agronomic use efficiency of N fertilizer (AE_N_) and recovery efficiency of N fertilizer (RE_N_) were significantly influenced by water and N regime, planting density and their interaction in both seasons (Figure 5), except for NUE_G_ in 2022–2023 (Figure 5A). In 2023–2024, DF increased the average NUE_G_ by 7.8%. Across both seasons, NUE_G_ tended to decline progressively with increasing planting density, particularly at high densities (PD6–PD8).

Averaged across planting densities, DF increased AE_N_ by 26.7–30.9% relative to CM (Figure 5C,D). AE_N_ exhibited a unimodal response to planting density, increasing from low to moderate densities and declining sharply at excessive densities (PD7–PD8). Under DF, AE_N_ reached peak values at intermediate densities (PD3–PD5), whereas under CM it declined markedly beyond moderate densities. At the highest density, AE_N_ dropped substantially under both management regimes, but the reduction was more pronounced under CM.

DF improved the averaged RE_N_ by 33.9–42.3% compared with CM (Figure 5E,F). RE_N_ increased from low to moderate densities and then decreased at high densities. DF consistently enhanced RE_N_ across densities, with the greatest improvement observed at intermediate densities. Under CM, RE_N_ declined sharply at high PD, indicating reduced nitrogen recovery efficiency under excessive canopy competition.

### 2.7. Correlation Analysis

**Figure 6:**
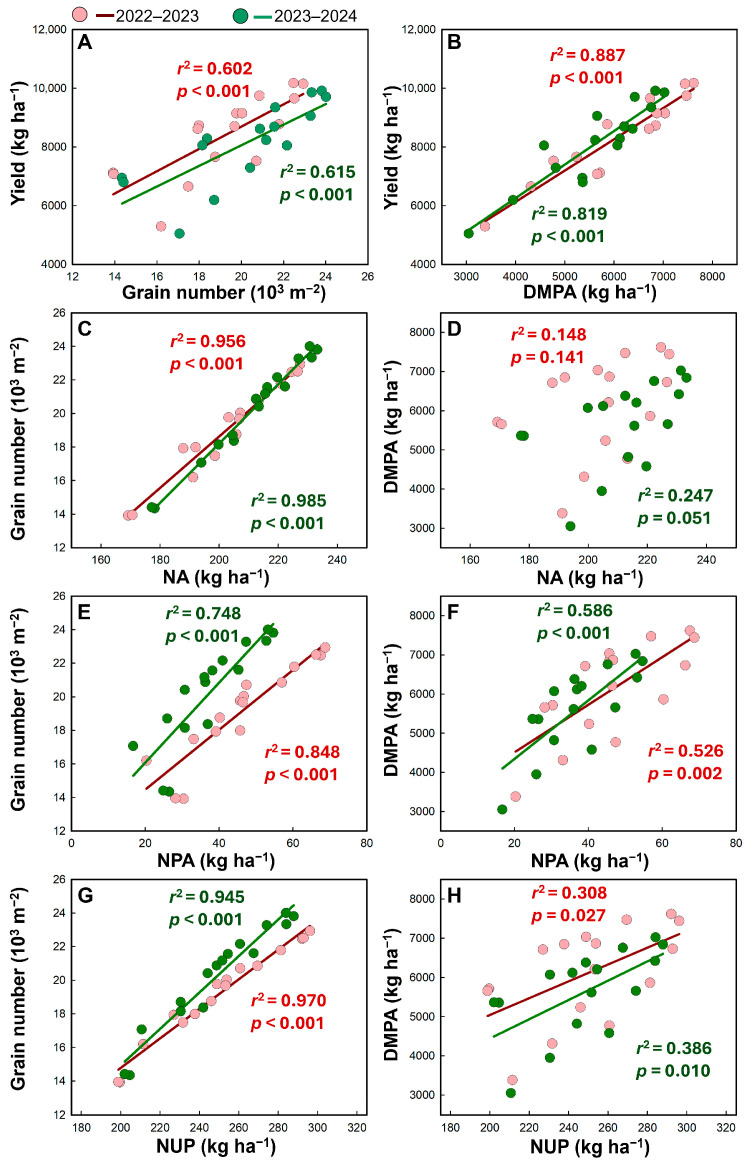
Relationships among grain yield, grain number, post-anthesis dry matter production (DMPA), N uptake at anthesis (NA), post-anthesis N uptake (NPA), and total nitrogen uptake at maturity (NUP) across planting densities and water–nitrogen management regimes in the 2022–2023 and 2023–2024 growing seasons.

Grain yield was positively correlated with grain number in both seasons (2022–2023: *r*^2^ ≥ 0.602, *p* < 0.001, Figure 6A). A stronger association was observed between yield and post-anthesis dry matter production (*r^2^* ≥ 0.819; *p* < 0.001, Figure 6B). N uptake at anthesis, after anthesis and at maturity were tightly associated with grain number (*r*^2^ ≥ 0.748, *p* < 0.001, Figure 6C,E,G), whereas their relationship with post-anthesis dry matter was not statistically significant (*r*^2^ ≤ 0.247, *p* > 0.05, Figure 6D) or relatively weak (*r*^2^ ≥ 0.308, *p* < 0.05, Figure 6F,H).

## 3. Discussion

Across two growing seasons, DF consistently outperformed CM in grain yield, producing 15.4–20.8% higher yields (Figure 1), in agreement with previous reports [18,22,23]. In practice, identifying an appropriate planting density (PD) range is essential for achieving high and reliable winter wheat yields [7,8,16,24]. In the present study, grain yield under both water–N regimes exhibited a typical quadratic response to PD, increasing from low densities to an optimum and then declining at excessive densities. Notably, DF shifted the optimum PD upward (456–487 seeds m^−2^) relative to CM (377–378 seeds m^−2^) and sustained high yields across a broader density interval (particularly PD3–PD6). This widened high-yield plateau suggests that DF reduced yield penalties at both low and high planting densities, thereby broadening the range of planting densities over which high yield could be maintained.

The yield advantage of DF was initially reflected in improved stand structure and sink formation. DF reduced basal N input, which likely constrained excessive tillering at high densities and prevented the overproduction of non-productive stems. Meanwhile, split N applications under DF improved synchronization between N supply and crop demand during key developmental stages (regreen-up, heading, and anthesis), thereby enhancing tiller survival and ear formation. As a result, increases in productive ear number were achieved without a proportional penalty in grains per ear (Table 1). Consistent with this interpretation, grain number per m^2^ (sink size) peaked at PD4 under CM and decreased significantly at PD6, whereas under DF it peaked at PD5 or PD6. Thus, DF facilitated the establishment and maintenance of a larger sink size at medium-to-high densities, creating a stronger basis for higher yield potential.

However, high yield depends not only on sink size but also on whether the crop can sustain adequate assimilate supply to fill that sink. Achieving high crop yield is widely recognized to require improved source–sink coordination, i.e., concurrent strengthening of assimilate supply and sink capacity [25,26,27]. In this study, DF increased post-anthesis dry matter accumulation by 15.5–17.6% relative to CM, and yield was strongly associated with post-anthesis dry matter production (*r*^2^ ≥ 0.819). These results are consistent with the hypothesis that, once a relatively large sink had been established under DF, achieving high yield at medium-to-high planting densities was associated with greater post-anthesis dry matter production during grain filling.

This source advantage is particularly important because high planting density commonly accelerates canopy closure and increases LAI, but it also intensifies self-shading and competition for N, shortening the effective photosynthetic duration and reducing canopy photosynthetic efficiency during grain filling [16,28,29,30]. Under CM, these density-induced constraints likely contributed to the decline in post-anthesis biomass production at excessive densities, consistent with the observed yield reduction beyond the optimum PD. In contrast, DF partly alleviated these limitations by sustaining a stronger post-anthesis source. DF maintained higher N nutritional status at anthesis (higher NNI across densities, Figure 4C,D), which likely improved leaf N concentration and delayed premature senescence. More importantly, DF markedly enhanced post-anthesis N uptake (by 47.7–49.5%). This pattern is consistent with the hypothesis that greater N uptake after anthesis may help sustain canopy greenness and photosynthetic activity during grain filling, particularly under relatively dense stands where N dilution is more pronounced. The significant correlations between post-anthesis N uptake and both sink size (*r*^2^ ≥ 0.748) and source capacity (*r*^2^ ≥ 0.526) further suggest that post-anthesis N acquisition under DF may have contributed to both sink realization and source support during grain filling. However, these relationships should be interpreted as associations rather than direct proof of a causal mechanism.

From a classical crop growth perspective, grain yield is determined not only by dry matter production but also by the efficiency with which assimilates are partitioned to grain, commonly quantified as harvest index [5,31,32]. Therefore, increased post-anthesis assimilation contributes to yield only when a sufficient proportion of the additional biomass is allocated to developing grains. In the present study, DF improved harvest index by 4.5–9.7%, indicating that the extra assimilates produced during grain filling were more effectively converted into grain rather than retained in vegetative tissues. This enhanced partitioning complements the DF-induced increases in sink size and post-anthesis source strength, providing an additional explanation for superior yield performance at medium-to-high densities.

Taken together, Figure 3 provides an interpretive framework for understanding why the DF management package was associated with higher yield and a broader high-yield planting density range. Compared with CM, DF was associated with a larger sink, reflected by a higher grain number per m^−2^ maintained at intermediate-to-high planting densities (with the peak occurring at PD5–PD6 rather than PD4 and without the marked decline observed at PD6 under CM), and with greater post-anthesis dry matter production, which is consistent with stronger source support during grain filling. These concurrent responses placed more DF treatments within the domain characterized by relatively high grain number together with relatively high post-anthesis dry matter accumulation, particularly across PD3–PD6, whereas CM more often showed patterns consistent with sink limitation at lower densities and source limitation or co-limitation at excessive densities. Improved N uptake, especially after anthesis, may have contributed to this pattern by helping to sustain canopy function during grain filling and support assimilate supply for sink realization. As a result, DF was associated with a broader planting density range over which near-maximum yield could be maintained, thereby reducing yield penalties when planting density deviated from the apparent optimum. Because DF involved simultaneous changes in irrigation method, irrigation frequency, and N application strategy, these responses should be interpreted as the integrated effect of the DF treatment package rather than the isolated contribution of any single component.

The improvements in REN and AEN further support the agronomic advantages of the DF management package. Low REN (often around or below 30%) has been widely recognized as a major constraint on yield improvement in intensive cereal systems in China [33,34,35]. In the present study, RE_N_ under CM reached only 31.2–31.5% at best (PD4), whereas DF increased peak RE_N_ to 41.4–42.9% (PD5). This pattern suggests that DF was associated with better synchronization between N supply and crop demand, possibly by maintaining more favorable root-zone N availability during periods of high N requirement, such as stem elongation to anthesis and early grain filling. By reducing the temporal mismatch between early-season N supply and later crop demand, DF may have enhanced crop N capture and reduced the fraction of fertilizer N exposed to loss pathways, thereby increasing the proportion of applied N recovered in aboveground biomass. Notably, the shift in peak REN from PD4 under CM to PD5 under DF suggests that DF was associated with more effective fertilizer N recovery at a relatively higher planting density, a pattern consistent with the higher canopy N status and greater post-anthesis source activity observed under DF.

AE_N_ reflects the marginal yield response to applied N, and conceptually its improvement is often associated with greater N recovery because yield gains depend, at least in part, on the amount of applied N taken up by the crop [36]. In this study, DF increased AE_N_ by 26.7–30.9% and simultaneously raised RE_N_ by 33.9–42.3% relative to CM, suggesting that the AE_N_ advantage under DF was closely associated with improved N recovery. Higher REN under DF is also consistent with better temporal matching between N availability and crop demand, which may have helped sustain canopy function during grain filling, in line with the greater post-anthesis N uptake observed under DF. The unimodal response of AEN to planting density further suggests that intermediate densities favored greater yield return per unit N input, whereas excessive densities likely intensified competition and N dilution, thereby limiting the conversion of recovered N into additional grain yield. Overall, these results demonstrate that DF enhanced both sink formation and post-anthesis source strength through improved N uptake dynamics, thereby enabling higher yield and reduced sensitivity to planting density variation, with intermediate densities (PD3–PD6) representing a practical range for achieving high yield alongside improved fertilizer-N effectiveness.

## 4. Materials and Methods

### 4.1. Site Description

**Figure 7:**
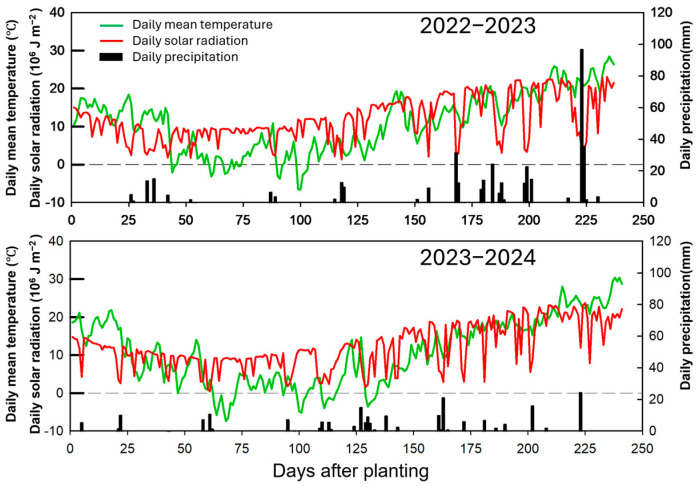
Daily mean temperature, solar radiation, and precipitation in 2022–2023 and 2023–2024 growing seasons.

Two consecutive field trials were carried out during the 2022–2023 and 2023–2024 winter wheat seasons at Quanyuan Town, Tancheng County, Shandong Province, China (34°42′ N, 118°25′ E). The local production system is dominated by a winter wheat–summer maize rotation. Seasonal precipitation totaled 407.9 mm in 2022–2023 and 221.9 mm in 2023–2024 (Figure 7). The experimental soil was classified as a clay loam. Prior to planting, soil samples (0–20 cm) were collected in each season to characterize baseline fertility. Soil organic matter was 16.0 and 16.6 g kg^−1^, total N was 1.02 and 0.98 g kg^−1^, Olsen-P was 16.36 and 88.04 mg kg^−1^, available K was 142.3 and 139.0 mg kg^−1^, and soil pH was 8.18 and 8.22 in 2022 and 2023, respectively.

### 4.2. Experimental Design and Crop Management

The experiment followed a split-plot arrangement with four replications. Water and N regime (WN) was treated as the main-plot factor, and planting density was treated as the subplot factor. Individual subplots measured 7.5 m × 1.6 m and consisted of eight rows (row spacing 0.20 m). The high-yielding winter wheat cultivar ‘Linmai 9’ was used. Eight target seeding rates were established: 100, 200, 300, 400, 500, 600, 700, and 800 seeds m^−2^ (PD1–PD8). Seeds were planted manually at approximately 3 cm depth. Planting dates were October 17 (2022) and October 14 (2023). Stand counts at the three-leaf stage indicated high and uniform establishment, with plant-to-seed ratios of 95.2% (2022) and 95.0% (2023), and no significant differences among treatments within each season.

Basal phosphorus and potassium were applied uniformly to all plots before sowing: 120 kg P_2_O_5_ ha^−1^ as calcium superphosphate (16% P_2_O_5_) and 90 kg K_2_O ha^−1^ as potassium chloride (52% K_2_O). The total N rate was 210 kg N ha^−1^ (urea, 46% N) under both WN regimes, but N timing differed. In CM, 60% of N (126 kg ha^−1^) was incorporated as a pre-planting basal application and the remaining 40% (84 kg ha^−1^) was applied once at jointing.

Under the DF regime, basal N application accounted for 40% of the total N rate (84 kg ha^−1^), while the remaining 60% was supplied through fertigation in four split applications at green-up, jointing, booting, and anthesis, accounting for 10%, 30%, 10%, and 10% of the total N input, respectively (21, 63, 21, and 21 kg ha^−1^).

Irrigation schedules also differed between the two management regimes. CM plots were flood-irrigated three times during the growing season, namely before winter dormancy, at jointing, and at anthesis, with 80 mm applied at each event. In contrast, DF plots were irrigated through a drip irrigation system six times, namely before winter dormancy, at green-up, jointing, booting, anthesis, and early milk stage, with 40 mm applied at each event. Thus, the two regimes differed not only in the timing of N application, but also in irrigation method and irrigation frequency, while receiving the same total irrigation amount over the season.

Drip tapes (16 mm in diameter) were installed at 0.40 m spacing, with one tape serving two wheat rows. Emitters were spaced 0.30 m apart and had a nominal discharge rate of 1 L^−1^. During fertigation, urea was dissolved in water and injected into the irrigation line using a Venturi injector before being distributed to each plot. Each fertigation event lasted approximately 6 h: fertilizer injection began during the second hour and continued for about 3 h, followed by flushing with clean water to clear the lines.

To calculate fertilizer N recovery efficiency (RE_N_) and agronomic efficiency (AE_N_), additional unfertilized plots corresponding to all eight planting densities under both water-management regimes were established in an adjacent field with similar soil conditions.

All plots were managed to avoid confounding stress effects. Disease and insect pressure were controlled as needed, and weeds were suppressed by herbicide applications conducted three times in each growing season.

### 4.3. Sampling and Measurements

The maximum stem number (main stems plus tillers) was recorded at jointing. Counts were taken in four representative central rows over 1.25 m length (equivalent to 1.0 m^2^). At maturity, the number of ears per unit area was determined, and the productive stem percentage was calculated as the ratio of the ears number at maturity relative to the maximum number of stems at jointing.

Leaf area and canopy development were assessed at anthesis. Plants were sampled from a representative central row over 0.50 m length (0.100 m^2^). Green leaf blades were separated and their area measured using a leaf area meter (LI-3100C, LI-COR, Lincoln, NE, USA). Leaf area index (LAI) was computed as

(1) LAI=Leaf area/Sampled land area

For biomass and plant N determination, whole aboveground plants were collected at anthesis and maturity from a central row segment of 0.50 m (0.100 m^2^) in each plot. At anthesis, samples were partitioned into stem (including sheath), leaf blade, and ear; at maturity, samples were separated into straw and grain. All plant fractions were heat-killed at 105 °C for 30 min and then oven-dried at 80 °C to constant weight for dry matter determination. Tissue N concentration (mg g^−1^) was measured using an automated Kjeldahl analyzer (Kjeltec 8400, FOSS, Hillerød, Denmark). Nitrogen uptake at anthesis (NA) and at maturity (NUP) was calculated as the sum of the products of dry matter and N concentration for each plant component. Post-anthesis N uptake (NPA) was calculated as the difference between NUP and NA. Based on yield and N uptake data, N use efficiency for grain production (NUE_G_), AE_N_ and RE_N_ were calculated as follows:

(2) $${NUE}_{G}(kg{kg}^{-1})=Yield/N\;uptake$$

(3) $${AE}_{N}(kg{kg}^{-1})=\left(Yield-Y_{0}\right)/N\;rate$$

(4) $${RE}_{N}\left(\%\right)=\left(N\;uptake-N_{0}\right)/N\;rate\times 100$$

where N uptake is the total aboveground N uptake at maturity, Y_0_ and N_0_ are yield and N uptake from the zero-N plots; and N rate is the applied N rate.

N nutritional status at anthesis was evaluated using the nitrogen nutrition index (NNI), which compares the measured canopy N concentration with the critical N concentration required to avoid N limitation. Specifically, NNI was defined as the quotient of the actual aboveground N concentration (N_t_) and the critical N concentration (N_ct_):

(5) $$NNI=N_{t}/N_{ct}$$

The critical N concentration (N_ct_) was obtained from the wheat critical N dilution relationship proposed by Justes et al. [37], expressed as a function of aboveground dry matter (DM) at anthesis:

(6) $$N_{ct}=5.35\times {DM}^{-0.442}$$

The actual N concentration at anthesis (N_t_) was calculated from the measured aboveground N uptake and dry matter at anthesis:

(7) $$N_{t}=NA/DMA$$

where NA is N uptake at anthesis (kg ha^−1^), and DMA is dry matter at anthesis (kg ha^−1^).

At maturity, grain yield was determined by harvesting ears from the center of each plot over 2.0 m^2^ (2.0 m length across five representative rows). Grain yield was converted to a standard moisture content of 13.0%, with moisture measured using a digital tester (PM8188A, Kett Electric Laboratory, Tokyo, Japan). Grain weight was determined from three randomly selected 50.00 g subsamples; grain weight values were also corrected to 13.0% moisture. Grains per ear (GPE) were calculated as

(8) $$GPE=Yield/\left(GW\times EN\right)\times 1000$$

where Yield is grain yield per unit area (g m^−2^), GW is single-grain weight (mg), and EN is ear number per m^2^.

### 4.4. Data Analysis

Statistical analyses were performed with Statistix 9.0 (Analytical Software, Tallahassee, FL, USA). Data were analyzed separately for each growing season using analysis of variance (ANOVA) appropriate for a split-plot design, with water–nitrogen management regime (WN) as the main-plot factor and planting density (PD) as the subplot factor. The interaction between WN and PD was also tested within each growing season, and the corresponding error terms for the main plot and subplot were used to assess the significance of main effects and interactions. When ANOVA indicated significant effects, treatment means were compared using a single consistent post hoc procedure at the 0.05 probability level. Similarities in treatment responses between growing seasons were evaluated descriptively rather than through a combined multi-year ANOVA. Simple linear regression and quadratic curve fitting were used to quantify relationships between variables, and the coefficient of determination (*r*^2^) was reported. Graphical representations of data were produced using SigmaPlot 15.0 (Systat Software Inc., Point Richmond, CA, USA) and Origin 2021 (OriginLab Corporation, Northampton, MA, USA).

## 5. Conclusions

Across two growing seasons, drip fertigation (DF) increased winter wheat grain yield by 15.4–20.8% compared with conventional management (CM) across planting densities. Yield responded quadratically to planting density under both regimes, but DF shifted the optimal density upward (456–487 seeds m^−2^ vs. 377–378 seeds m^−2^ for CM) and maintained near-maximum yields across a wider density range (300–600 seeds m^−2^), indicating greater yield resilience. DF improved population productivity by increasing productive stem percentage and yield per ear, enhancing grain number per m^2^, and it promoted post-anthesis dry matter production and harvest index, reflecting stronger source capacity and better partitioning. DF also markedly increased post-anthesis N uptake (48–50%), leading to higher total N uptake and grain N accumulation, and it improved agronomic and recovery efficiencies of N use by 26.7–30.9% and 33.9–42.3%, respectively. Under the conditions of this study, DF was associated with a broader high-yield planting density range and smaller yield penalties when planting density deviated from the apparent optimum.

## Figures and Tables

**Table 1:** Ear number (EN), grains per ear (GPE), grain weight (GW) and grain number (GN) of winter wheat in the 2022–2023 and 2023–2024 growing seasons.

WN	PD	2022–2023				2023–2024			
	(m^−2^)	EN (m^−2^)	GPE	GW (mg)	GN (10^3^ m^−2^)	EN (m^−2^)	GPE	GW (mg)	GN (10^3^ m^−2^)
CM	100	359.2 g	38.8 a	50.8 a	13.9 f	367.0 g	39.3 a	47.2 a	14.4 g
	200	463.7 f	38.7 a	48.1 b	17.9 cd	474.1 f	38.3 ab	44.4 b	18.1 e
	300	552.3 e	35.8 b	46.3 c	19.8 a	568.0 e	36.8 b	41.3 c	20.9 bc
	400	620.7 d	32.3 c	45.7 c	20.0 a	630.4 d	34.2 c	40.3 d	21.6 a
	500	664.4 c	29.6 d	44.2 d	19.7 ab	680.6 c	31.1 d	38.9 e	21.2 ab
	600	694.3 b	27.0 e	40.9 e	18.8 bc	717.9 b	28.4 e	35.7 f	20.4 c
	700	729.9 a	24.0 f	38.1 f	17.5 d	749.3 ab	25.0 f	33.1 g	18.7 d
	800	745.9 a	21.7 g	32.7 g	16.2 e	775.1 a	22.0 g	29.6 h	17.1 f
	Mean	603.8 B	31.0 B	43.3 B	18.0 B	620.3 B	31.9 B	38.8 B	19.0 B
DF	100	366.5 g	38.0 a	51.1 a	13.9 e	375.8 g	38.2 a	48.4 a	14.3 d
	200	482.5 f	37.3 a	48.6 b	18.0 d	493.6 f	37.2 a	45.1 b	18.4 c
	300	580.3 d	35.9 b	46.7 c	20.9 c	600.8 e	36.0 b	43.3 c	21.6 b
	400	654.2 d	34.3 c	45.3 d	22.5 ab	667.3 d	35.0 b	42.2 d	23.3 a
	500	708.8 c	32.4 d	44.3 e	22.9 a	729.2 c	32.7 c	41.6 d	23.8 a
	600	751.6 b	30.0 e	42.9 f	22.5 ab	763.8 bc	31.4 d	40.4 e	24.0 a
	700	784.3 ab	27.8 f	40.3 g	21.8 b	797.7 ab	29.2 e	38.9 f	23.3 a
	800	800.4 a	25.9 g	36.4 h	20.7 c	817.3 a	27.1 f	36.3 g	22.2 b
	Mean	641.1 A	32.7 A	44.4 A	20.4 A	655.7 A	33.3 A	42.0 A	21.4 A
ANOVA	---------------------------------------------*p* value-------------------------------------------								
WN		0.001	0.003	0.004	0.001	0.001	0.009	<0.001	0.001
PD		<0.001	<0.001	<0.001	<0.001	<0.001	<0.001	<0.001	<0.001
WN × PD		0.252	<0.001	<0.001	<0.001	0.619	<0.001	<0.001	<0.001

Different uppercase letters indicate a significant difference between the means of water-N regimes (WN) across eight planting densities (PD) according to the least significant difference test (α = 0.05). Within a column and a specific WN, different lowercase letters indicate significant differences between planting densities according to the least significant difference test (α = 0.05).

**Table 2:** Dry matter production at anthesis (DMA), after anthesis (DMPA), at maturity (DM) and harvest index (HI) of winter wheat in the 2022–2023 and 2023–2024 growing seasons.

WN	PD	2022–2023				2023–2024			
		DMA	DMPA	DM	HI	DMA	DMPA	DM	HI
	(m^−2^)	(kg ha^−1^)			(%)	(kg ha^−1^)			(%)
CM	100	6668 e	5657 d	12,325 e	50.0 a	7153 e	5365 c	12,518 e	47.2 a
	200	8409 d	6712 b	15,120 c	49.6 a	9250 d	6070 b	15,320 c	45.7 ab
	300	10,034 c	7035 a	17,069 ab	46.6 b	10,669 c	6378 a	17,047 ab	44.0 b
	400	10,671 b	6867 ab	17,538 a	45.4 bc	11,321 abc	6206 ab	17,527 a	43.1 bc
	500	10,970 ab	6211 d	17,181 ab	44.1 c	11,739 a	5616 c	17,355 ab	41.3 c
	600	11,141 a	5236 e	16,377 b	40.7 d	11,751 a	4819 d	16,571 b	38.3 d
	700	10,997 ab	4313 f	15,310 c	37.8 e	11,474 ab	3949 e	15,423 c	34.9 e
	800	10,771 ab	3381 g	14,152 d	32.5 f	10,995 bc	3049 f	14,044 d	31.3 f
	Mean	9958 B	5676 B	15,634 B	43.3 B	10,544 B	5182 B	15,725 B	40.7 B
DF	100	6572 e	5713 c	12,285 f	50.4 a	7181 e	5357 e	12,538 e	48.2 a
	200	8497 d	6847 b	15,344 e	49.5 ab	9421 d	6118 c	15,539 d	46.4 ab
	300	10,360 c	7473 a	17,833 cd	47.5 bc	11,036 c	6756 a	17,793 bc	45.7 bc
	400	11,613 b	7618 a	19,231 ab	46.0 cd	12,106 b	7024 a	19,130 a	44.8 bcd
	500	12,232 ab	7441 a	19,673 a	44.9 de	12,695 a	6840 a	19,535 a	44.1 cd
	600	12,567 a	6732 b	19,300 ab	43.5 ef	12,751 a	6421 b	19,172 a	44.0 cd
	700	12,482 a	5862 c	18,345 bc	41.6 f	12,742 a	5656 d	18,399 ab	42.8 de
	800	12,159 ab	4772 d	16,931 d	38.7 g	12,347 ab	4578 f	16,925 d	41.4 e
	Mean	10,810 A	6557 A	17,368 A	45.3 A	11,285 A	6094 A	17,379 A	44.7 A
ANOVA	-------------------------------------------------*p* value-----------------------------------------								
WN		0.003	<0.001	0.002	0.038	<0.001	<0.001	0.001	<0.001
PD		<0.001	<0.001	<0.001	<0.001	<0.001	<0.001	<0.001	<0.001
WN × PD		<0.001	<0.001	<0.001	0.001	0.018	<0.001	<0.001	<0.001

Different uppercase letters indicate a significant difference between the means of Water and N regimes (WN) across eight planting densities (PD) according to the least significant difference test (α = 0.05). Within a column and a specific WN, different lowercase letters indicate significant differences between PD according to the least significant difference test (α = 0.05).

**Table 3:** N uptake at anthesis (NA), after anthesis (NPA), at maturity (NUP) and grain N accumulation (GNA) of winter wheat in the 2022–2023 and 2023–2024 growing seasons.

WN	PD	2022–2023				2023–2024			
		NA	NPA	NUP	GNA	NA	NPA	NUP	GNA
	(m^−2^)	(kg ha^−1^)				(kg ha^−1^)			
CM	100	170.6 e	28.2 d	198.8 d	149.7 d	177.2 d	24.9 c	202.1 c	147.3 d
	200	187.8 d	39.1 b	227.0 b	183.6 b	199.8 bc	30.7 b	230.5 b	175.1 b
	300	203.2 ab	45.7 a	248.9 a	193.7 a	212.5 a	36.3 a	248.8 a	185.5 a
	400	207.1 a	46.7 a	253.8 a	196.6 a	216.3 a	38.2 a	254.5 a	191.4 a
	500	206.9 ab	46.4 a	253.3 a	191.7 a	215.6 a	36.0 a	251.6 a	184.8 a
	600	205.8 ab	40.2 b	246.0 a	171.4 c	213.5 a	30.7 b	244.2 a	166.3 c
	700	198.6 bc	33.1 c	231.7 b	152.3 d	204.6 b	25.9 c	230.5 b	145.9 d
	800	191.2 cd	20.3 e	211.5 d	121.5 e	194.0 c	16.7 d	210.7 c	118.3 e
	Mean	196.4 B	37.5 B	233.9 B	170.1 B	204.2 B	29.9 B	234.1 B	164.3 B
DF	100	169.2 e	30.4 d	199.6 f	150.4 g	178.2 d	26.5 e	204.6 f	150.7 e
	200	192.0 d	45.7 c	237.7 e	186.2 e	205.0 d	36.9 d	241.9 e	180.1 d
	300	212.5 c	57.0 b	269.5 c	209.9 c	222.3 ab	45.3 b	267.5 cd	205.5 b
	400	224.6 a	67.5 a	292.1 a	221.5 a	231.3 a	52.8 a	284.1 ab	219.8 a
	500	227.4 a	68.7 a	296.1 a	222.8 a	233.2 a	54.7 a	287.9 a	221.4 a
	600	226.6 a	66.2 a	292.8 a	214.9 b	230.7 ab	53.2 a	283.9 ab	220.9 a
	700	220.9 ab	60.4 b	281.3 b	197.2 d	226.9 ab	47.3 b	274.2 bc	208.6 b
	800	213.3 bc	47.4 c	260.7 d	171.4 f	219.7 b	40.9 c	260.6 d	188.7 c
	Mean	210.8 A	55.4 A	266.2 A	196.8 A	218.4 A	44.7 A	263.1 A	199.5 A
ANOVA	------------------------------------------------*p* value--------------------------------------------								
WN		<0.001	<0.001	<0.001	<0.001	<0.001	<0.001	<0.001	<0.001
PD		<0.001	<0.001	<0.001	<0.001	<0.001	<0.001	<0.001	<0.001
WN × PD		<0.001	<0.001	<0.001	<0.001	0.006	<0.001	<0.001	<0.001

Different uppercase letters indicate a significant difference between the means of water-N regimes (WN) across eight planting densities (PD) according to the least significant difference test (α = 0.05). Within a column and a specific WN, different lowercase letters indicate significant differences between planting densities according to the least significant difference test (α = 0.05).

## Data Availability

The original contributions presented in this study are included in the article. Further inquiries can be directed to the corresponding author.
